# Additively manufactured magic cube platforms for fully integrated wireless sensing nodes for Internet of Things applications

**DOI:** 10.1038/s41598-023-49130-0

**Published:** 2023-12-08

**Authors:** Madeline Elaine Holda, Charles Lynch, Manos M. Tentzeris

**Affiliations:** School of Electrical and Computer Engineering, Georgia Institute of Technology, Atlanta, GA USA

**Keywords:** Engineering, Electrical and electronic engineering, Electronic devices

## Abstract

Wireless sensor networks for environmental monitoring are a key feature in developing the Internet of Things. Although there has been much research in developing components for wireless sensing nodes, advances in creating fully integrated sensing nodes is limited. Furthermore, because most sensing nodes that have been developed are intended to perform a fixed task, each new effort to design an integrated sensing node with different functionality must start from scratch. Here we introduce a broadly applicable platform for the development and production of fully integrated wireless sensing nodes. The platform is an additively manufactured cube that has different subsystems occupying separate faces of the 3D structure. While both additively manufactured sensors and cube-shaped wireless sensing nodes have been previously reported, these two approaches have yet to be combined. A key technology that enables this is the use of additively manufactured, nonplanar bent microstrips. This realization offers a “plug-and-play” approach to sensor node design, as the subsystems are considered modular and can be swapped to alter the function of the device. Implementing this concept enables the rapid development and deployment of wireless sensor networks.

A key feature for developing the Internet of Things (IoT) is real-time environmental monitoring. This can be accomplished by a wireless sensor network (WSN), which is composed of numerous sensor nodes distributed around an environment that report back to a controlling IoT network. These nodes can measure any type of environmental data that sensors have been developed for, including temperature^1,2^, humidity^2,3^, light intensity^4,5^, vibrations^6,7^, and gases^8^. Once the sensor nodes have communicated relevant information to the base station, the controlling IoT network evaluates this data and can take action if necessary^9–12^.

WSNs are critical in a variety of applications, including smart agriculture, smart homes, smart infrastructure, monitoring extreme environments, and smart manufacturing (Fig. 1a). One broader goal these systems can advance is environmental sustainability. For example, WSNs can be used in smart agriculture to monitor soil health and other environmental variables, allowing for more precise control over crop health^13,14^. This leads to larger crop yields without needing more resources, known as sustainable intensification. In the case of smart homes, WSNs can be used to reduce overall energy consumption via power scheduling^15^. Another goal of WSNs is to improve safety. With smart infrastructure, a WSN could monitor the physical integrity of commercial and private infrastructure^3,7^. By providing advance notice on structural weaknesses, WSNs could make infrastructure safer for humans. Furthermore, WSNs have the ability to monitor extreme environments^16,17^, allowing for increased control over these environments without needing to risk human lives. The optimizations possible with WSNs could prove to be a revolutionary technology in the coming decades^18^.

To practically implement a WSN, these sensor nodes must be small in size and low cost. Although many different fabrication processes can be used to create the nodes of a WSN, one that is of particular interest is additive manufacturing. This technology is advantageous for creating WSNs with many nodes because it is a reliable and scalable way to fabricate small, low-cost devices. Additively manufactured prototypes can be fabricated quickly and inexpensively, allowing for rapid prototyping^19^. Because additive manufacturing involves depositing material, rather than removing material from a larger sample as in subtractive manufacturing, it reduces waste^20^. It also allows for fabrication of custom geometries that are difficult to create using conventional techniques^21^.

Additive manufacturing is a highly versatile technology, and has already been used to fabricated a variety of components that could be used in a WSN sensing node. Additively manufactured sensors can measure a range of environmental variables, including pressure^22,23^, humidity^3,24^, temperature^25,26^, food spoilage^27^, gases^28^, and the presence of lead^29^. Additive manufacturing has also been used to create antennas^30,31^, RFID tags^28,32,33^, and RF backscatter frontend systems^31^. Because these components have been developed in isolation, research on integration is needed for them to be practically implemented in fully additively manufactured wireless sensing nodes.

These components perform many different functions, which creates a need for a single platform that can integrate these diverse subsystems. An appropriate solution must fulfil two requirements: Be standard enough so that all sensor nodes can be fabricated using a single process, and yet be flexible enough so that different sensor node designs can be developed for a wide range of applications. In this Article, we propose an integrated platform for wireless sensing nodes that offers a “plug-and-play” approach, in which different additively manufactured components can be selected and combined using a single fabrication process. This approach seeks to address the inadequacies of current integration methods.

This work presents a fully integrated, fully printed wireless sensor node for IoT applications. The sensor node described here serves as a proof-of-concept for the platform presented in this work, in which additively manufactured components occupy separate faces of a 1cm x 1cm x 2cm rectangular prism. The proof-of-concept design consists of four major components, all additively manufactured: a resistive temperature sensor, baseband circuit, backscatter frontend, and antenna. We propose that all of these components are considered modular elements, and can be swapped to meet a different design goal without the need to redesign the entire platform.

This unique 3D topology can only be realized if there is an RF transmission line that can connect components located on different faces of the device. These transmission lines must also be compatible with the additive manufacturing fabrication process used for the rest of the design. Although the properties of these lines is trivial when connecting DC components, accomplishing this with RF transmission lines is more nuanced. The proposed solution is a nonplanar bent microstrip trace, referred to as a “radial interconnect,” or simply “interconnect.” In this Article, we show that these fully printed radial interconnects are broadband and low-loss, which is essential for enabling the modular and adaptable attributes of the proposed sensing node platform.

Given the diversity of applications in which wireless sensing nodes can be implemented, the advantages of the “magic cube” platform over others is clear (Fig. 1b). The steps used to create the individual components of the device, such inkjet printing silver nanoparticle ink and attaching lumped element components with silver epoxy, are identical to those used for the fully integrated node. As a result, new components can be prototyped for the device without needing to develop a new fabrication process. Furthermore, this approach allows for direct printing and manufacturing of multiple sections of the device as a fully integrated, single unit. In this way, the platform and its “plug-and-play” approach allows for a greater variety of sensor nodes to be designed and produced than with previous methods, and at a faster pace of development.

**Figure 1:**
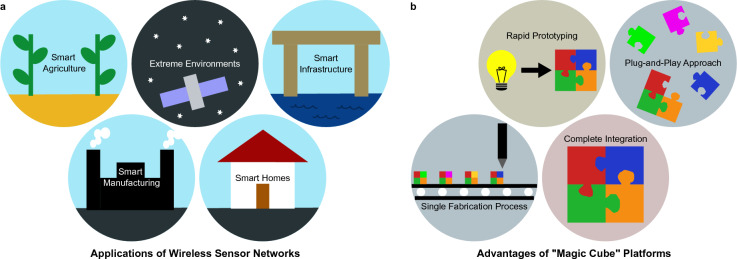
**The need for wireless sensor networks.** (**a**) Practical applications for wireless sensor networks. (**b**) Motivation for the modular cube platform.

## Fully integrated, fully printed cube platform

**Figure 2:**
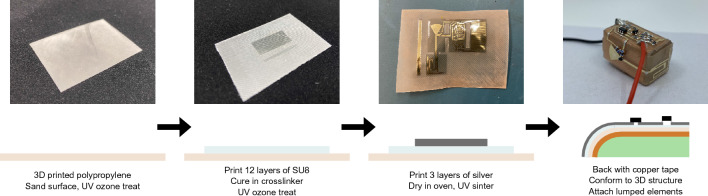
**“Magic cube” platform fabrication process.** A step-by-step illustration of the process used to fabricate the fully integrated cube platform.

The sensing device is additively manufactured as a fully integrated system, which has not been done in previous works. By inkjet printing conductive silver nanparticle ink, it is possible to produce a 2D pattern on a thin substrate, which is then conformed to a rigid 3D printed structure. The approach presented here takes advantage of both 2D and 3D printing to create a fabrication process that is rapid, reliable, and repeatable. The fabrication process is illustrated in Fig. 2, with further details provided in Methods.

This unique approach allows all major components of the device to be fabricated together as a single system, rather than making everything separately and joining the pieces together during post-fabrication. For example, the process used to produce an additively manufactured sensing node for detecting space orbital debris required individual components to be manufactured separately using different techniques, which were later combined^17^. In contrast, the system presented here offers complete integration of all system components during printing, which simplifies the fabrication process. More importantly, a single fabrication setup can be used for different sensor nodes because it is possible to fabricate components beyond the ones developed here.

Other fabrication techniques for creating cube-shaped wireless sensor nodes have been previously reported. A modular stack of PCBs, each having a separate function, which can then be swapped to meet different design goals was reported in^34^. However, the fabrication process is extremely complex, requiring a multi-layer PCB with ball grid array solder connections. Other works place circuitry on the outside of the cube, reducing fabrication complexity. However, not all of these sensor nodes are modular, which limits their potential applications to the specific tasks they were originally designed to perform^35^. While this can be avoided by designing a multi-functional sensor node, doing so comes at the cost of increased complexity and vestigial subsystems^36^. A modular, cube-shaped wireless sensing node was developed in^37^, but this system uses a battery. Since batteries have a finite lifetime, they require periodic replacement for continued device operation. This leads to increased maintenance costs and decreased physical robustness for a system. Battery-free devices, such as the one presented here, avoid these issues by using a power source that does not require replacement.

Additionally, these systems do not take advantage of additive manufacturing techniques. As discussed in the previous section, benefits of additive manufacturing include its low cost and support of rapid prototyping. One example of an additively manufactured, cube-shaped platform is a buoyant Lagrangian sensor^38^. However, as this sensing node does not have modular components, it can only be used for the niche application of flood monitoring. Another example is an energy harvesting 3-D printed origami packaging structure^39^. But again, this sensing node does not have modular components, and therefore does not enjoy the same benefits as the platform presented here.

The versatility of the platform is enabled by having modular components and using additive manufacturing to fabricate these components. Modifying the design file of a component allows it to be immediately printed with a new function, without affecting the rest of the design. For example, the temperature sensor could be swapped with another resistive sensor to detect a different environmental variable. Although the antenna presented in this design is omnidirectional and operates at 5.8 GHz, it could be replaced with a directional antenna, or one operating at a different frequency. As a third example, the solar cell used as a power source in this system could be replaced with another power source, such as a piezoelectric material or an energy-harvesting subsystem. The ability to leverage multiple battery-free power sources is particularly significant given the importance of battery-free technologies in developing wireless sensing nodes^40^.

The modular feature of the platform drastically broadens its range of potential applications. The architecture presented here is not limited to temperature detection, but could be used to monitor an entirely different environmental variable. It is not limited to receivers operating at 5.8 GHz, as the antenna could also be modified. The device could be used in areas with insufficient light to power the solar cell if another battery-free power source is used. It is also possible to incorporate components that add entirely new functions, such as one that adds a fixed identification metric to the backscatter modulation pattern, allowing for individual sensing nodes to be distinguished. The only limitation is fitting everything onto the six faces of the cube.

In this way, a single wireless sensor network with many sensor nodes, each serving a different function, could be created using a single platform and fabrication process. This approach enables scalability because of its simplicity, versatility, and use of additive manufacturing. By definition, WSNs require a large number of sensor nodes, so scalability is a key factor for the development and eventual deployment of practical WSNs, which may require hundreds or thousands of sensor nodes. Furthermore, additive manufacturing is generally low-cost, another requirement of highly scalable systems. The modularity of the platform does not compromise its miniaturization. The device presented here as a proof-of-concept is 1cm x 1cm x 2cm in size and weighs only 5.16 g, with the solar cell weighing an additional 1.8 g. Small sensing nodes are critical for a practical WSN, as the presence of sensor nodes cannot interfere with typical operations in the environment being monitored. Finally, the device is low-power, with a typical operating voltage of 3 to 3.4 V and a current draw of around 10 mA.

As with any other approach, there are certain downsides to the one presented here. Once a sensing node is fully fabricated, it cannot be modified, as would be the case with designs involving microprocessors and programmable firmware. Nevertheless, the approach proposed in this paper allows for the seamless function modification design and implementation by taking advantage of the modularity feature, just modifying the printed topology and materials. Additionally, although the power consumption is on the order of milliwatts, some applications may be more suited for microwatt power consumption. That said, microwatt power consumption could be achieved by redesigning the baseband circuit and backscatter frontend accordingly.

## Form factor and radial interconnects

Practically implementing this modular, highly adaptable platform requires a means of connecting components on separate faces of the device with RF transmission lines. The proposed solution, briefly described in the Introduction, is a nonplanar bent microstrip trace known as a “radial interconnect,” or simply “interconnect.” Although placing all RF circuitry on the same face of the device would eliminate the need for radial interconnects, doing so would place additional constraints on the design. By forcing all RF components to occupy a single face of the cube, the total space available for these components is significantly reduced. It would also limit the modularity of the platform by only allowing for one RF component to be swapped.

Microstrip lines are a vital RF technology, having been extensively studied and characterized for decades^41^. However, existing research on curved microstrip lines is limited to studying lines bent such that the overall structure remains planar, with the microstrip snaking across a planar dielectric surface. These bends radiate electromagnetic energy, and are therefore lossy^42–44^. The work presented here looks at a microstrip line that curves between two right-angle spatial planes, which results in a nonplanar structure. To date, no prior research has been found on this topic. It can be assumed that, as with their planar counterparts, transmission lines curved in this way will radiate, and will therefore have additional loss as compared to a flat transmission line. This is because any nonuniform structure carrying an RF signal, such as a bend, will radiate electromagnetic energy^45^.

**Figure 3:**
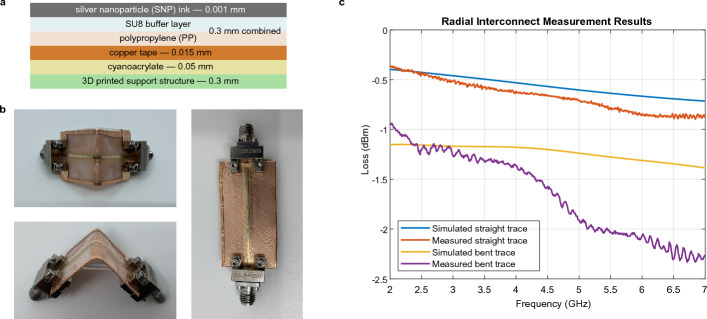
**Radial interconnects. **The interconnects provide RF connections between faces of the 3D cube. (**a**) Complete stackup of a radial interconnect, with thicknesses labeled. (**b**) Images of the radial interconnect and a straight trace. (**c**) Simulated and measured loss associated with radial interconnect and straight trace of length 43 mm, with 0.35 dB of connector loss removed from measured results.

The radial interconnects were first developed and characterized in isolation, before incorporation with the rest of the device. Prior to fabrication, the radial interconnects were simulated in CST Microwave Studio. The fabrication process outlined in the previous section is used, with copper tape adhered to the bottom of the substrate to serve as a conductive ground plane for the microstrip line. The line is then carefully folded over a rigid, 3D printed 90° bend, secured with adhesive, and attached to end launch connectors for testing. A complete stackup is show in in Fig. 3a, and images of the radial interconnects and a straight trace are shown in Fig. 3b.

Overall, the simulation and measurement results show that the printed 90° interconnects are low-loss and broadband. Connector loss was calculated to be 0.175 dB per connector, for a total of 0.35 dB for any trace with two connectors, and this is removed from the measured data shown in Fig. 3c. The overall transmission line loss for a straight trace is 0.0356 dB/m, which is incredibly low. Although bending the transmission lines does increase loss by approximately 0.5  to 0.1 dB, the total loss remains low and relatively flat across the 2  to 7 GHz frequency range that was measured. Differences between simulation and measurement results are due to imperfections in the fabrication process. The measured bent trace exhibits a greater loss per unit length from 3.5 to 4.5 GHz than the simulated bent trace. This is believed to be caused by defects introduced during the bending step of the fabrication process, which are not present in the simulation model or straight trace, and which lead to frequency-dependent loss.

Applications of radial interconnects extend beyond their use in the wireless sensor node platform presented in this Article. These interconnects can be used to connect two or more RF components existing on separate faces of any fixed 3D structure, which potentially allows other 2D designs to be conformed to a 3D structure, reducing the overall footprint of the design. Furthermore, these interconnects could be used in non-cube geometries, provided that the bending angle is no more than 90°. It is reasonable to assume that the performance of such a microstrip line would fall between the performances of the straight microstrip trace and 90° bent microstrip trace. Further research on this topic could characterize bending angles greater than 90°, which would further expand the possible geometries for these radial interconnects.

## System components and development

**Figure 4:**
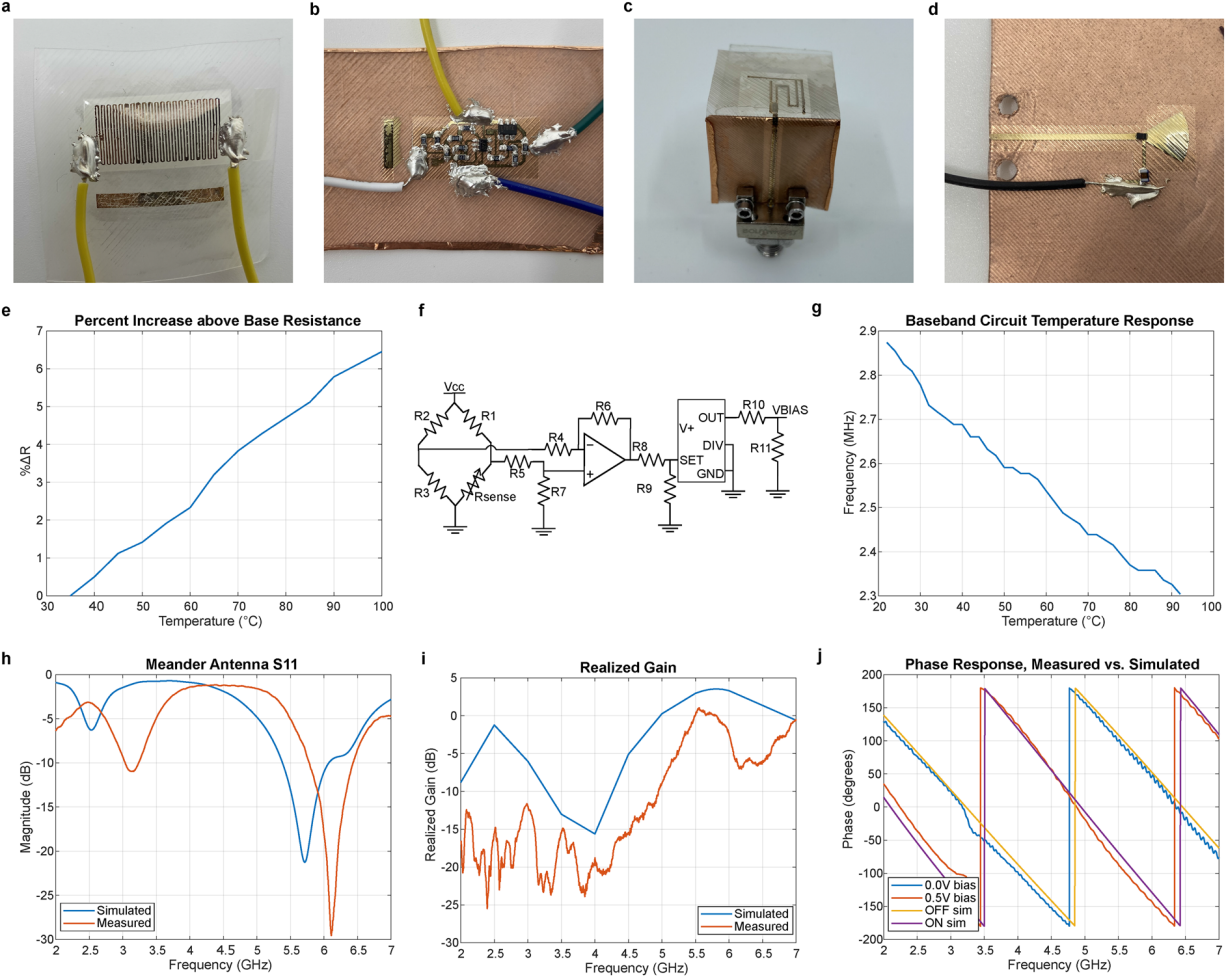
**Development of individual components to be integrated with the “magic cube” platform.** (**a**–**d**) Components developed and characterized in isolation: temperature sensor (**a**), baseband circuit (**b**), antenna (**c**), and backscatter frontend (**d**). (**e**) Temperature sensor percent increase above base resistance. (**f, g**) Schematic of baseband circuit (**f**) and its output frequency response to temperature (**g**). (**h, i**) Comparison of simulated and measured antenna $S_{11}$ parameters (**h**) and realized gain (**i**). (**j**) Phase response of backscatter frontend over frequency range.

There are four main components to the system, each located on a separate face of the rectangular prism: the resistive sensor (Fig. 4a), baseband circuit (Fig. 4b), antenna (Fig. 4c), and backscatter frontend (Fig. 4d). These components were fabricated and characterized separately prior to integration, which is discussed in the final section. The steps taken to develop and integrate the components demonstrate the modular design process that we envision for the cube platform.

The sensing element used in this design is a temperature sensor, which could be replaced by any sensor that shows a change in resistance in response to some environmental variable. The temperature sensor is a resistive temperature detector (RTD), implemented by a long, thin trace which is inkjet printed using silver nanoparticle ink^25^. Silver has a positive temperature coefficient of resistance, so its resistivity increases with temperature^46^. The resistance of the temperature sensor was recorded as it was heated from room temperature to 100°C. The percent increase above base resistance was then calculated (Fig. 4e), with the estimated average sensitivity calculated to be 0.0993 $\%\Delta$R/°C.

The baseband circuit is responsible for producing an oscillating signal with a frequency that varies with respect to the resistance of the sensor. In this design, its active components are powered by a solar cell, but as previously discussed, the modular cube platform allows for the solar cell to be replaced with any other power source that meet the size requirements of the device. The baseband circuit, a schematic of which is shown in Fig. 4f, has three main components: a Wheatstone bridge, an operational amplifier configured in differential mode, and a voltage controlled oscillator (VCO). Resistor R1 is selected depending on the base resistance of the sensor, which in this case occurs at room temperature. The voltage at the point between R1 and Rsense is around 400 mV, and this value increases with increasing sensor resistance. The baseband circuit was connected to the temperature sensor using wires, and the frequency response was recorded as a function of temperature, with results shown in Fig. 4g. Note that a different resistance value for the temperature sensor will result in a different initial output frequency.

The antenna communicates with the interrogator, reflecting an incident signal back to the source. The antenna is fabricated such that it is fed from a radial interconnect. This particular meander antenna^47^ fits onto a 1cm x 1cm area and operates at 5.8 GHz. The $S_{11}$ results in Fig. 4h show a simulated return loss of 18.07 dB and a measured return loss of 10.55 dB. The realized gain results are shown in Fig. 4i, with a simulated realized gain of 3.55 dB and a measured realized gain of 0.091 dB.

The RF backscatter frontend modulates the radar cross-section (RCS) of the device by switching the diode, allowing for tag information to be transmitted to the reader via the difference in load states^48^. The backscatter frontend consists of a diode driven by the temperature-sensitive oscillating signal generated by the baseband circuit. This system uses a phase shift keying (PSK) modulation scheme, which is ideal for low-power applications^49^, so the phase response of the backscatter frontend for the diode ON and OFF states must be 180° apart. Simulated and measured results demonstrating this phase shift are shown in Fig. 4j.

## Leveraging the cube platform for integration

The advantage of the platform presented in this Article is that the faces of the cube are occupied by modular subsystems, offering a “plug-and-play” approach in which individual components can be swapped to alter the function of the device. In this section, we present two types of integration tests that can be performed prior to fabricating the fully printed system. The first is the fabricated integration of the antenna, backscatter frontend, and radial interconnect to form an RF tag, and the second is a component-wise integration of previously fabricated subsystems, in which the individually fabricated components are joined with wires and tested. These tests serve as a proof-of-concept for the modular development platform presented here.

**Figure 5:**
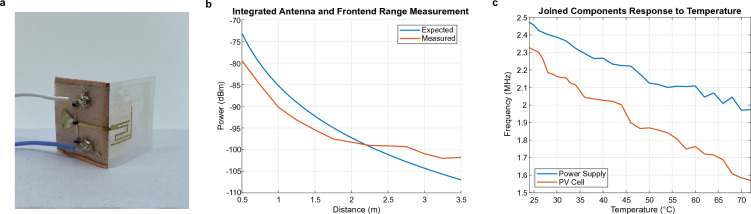
**Integration results. **(**a**) Integration of the backscatter frontend, radial interconnect, and antenna to create an RF tag. (**b**) Expected and measured results for the range test. (**c**) Modulation frequency response to temperature for individual components joined with wires.

To integrate the antenna, backscatter frontend, and interconnects, the three components were fabricated as a single unit, shown in Fig. 5a, to form an RF tag. The antenna and frontend are placed on 90° spatial planes and joined by a radial interconnect, which is the same configuration used in the final device. The differential RCS^50^ of the tag was measured at 5.8 GHz, which had an expected value of $-$33.02 dBsm and a measured value of $-$33.29 dBsm. A ranging test was also performed, with good agreement between expected and measured results, shown in Fig. 5b.

In order to test whether a particular configuration is viable, individually fabricated components are connected with wires and tested as a complete system. In an industrial-scale realization of this platform, an inventory of verified components could be created, from which parts could be selected as needed and tested in the way described here. After this quick verification is complete, all the designers would have to do is print and assemble the integrated device.

The baseband circuit and temperature sensor were connected to the integrated RF tag, and the modulation frequency response was measured with respect to temperature, shown in Fig. 5c. To further emphasize the modular aspect of the platform, the test was performed twice, once with a power supply and once with a solar cell. Because the voltage provided by the solar cell was slightly higher than the power supply, 3.4 V instead of 3 V, the overall modulation frequency was lower when the system was tested with the solar cell. As expected, the modulation frequency decreases with increasing temperature. The response is less monotonic that that of the isolated baseband circuit and temperature sensor. This is because the inclusion of the RF tag increases the complexity of the system, which adds noise to the data.

**Figure 6:**
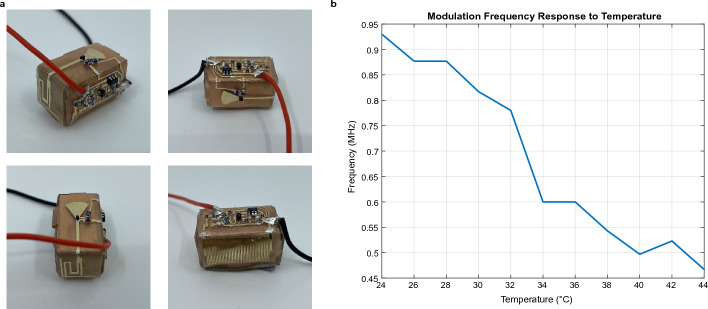
**The fully integrated device. **(**a**) Photographs of the complete device, integrated using the cube platform. (**b**) Temperature response of the device modulation frequency.

With the design verification complete, the fully integrated cube platform is used to fabricate the device, where all components are printed together as a complete system. The final device is shown in Fig. 6a. The modulation frequency of the device is measured as the temperature is increased from 24 to 44°C. Due to the physcial configuration of the measurement setup, the temperature of the device cannot be raised above 44°C. However, based on the results in Fig. 5c, it is reasonable to believe that the device could function at higher temperatures, up to at least 72°C. The final results in Fig. 6b show that the device modulation frequency decreases with increasing temperature, as expected. The sensitivity of the fully integrated system was calculated to be $-$25.2 kHz/°C, which can be set by adjusting the resistor values of the Wheatstone bridge and operational amplifier appropriately. The response of the fully integrated device shows more noise as compared to the joined components test because of the increased complexity of the system.

## Conclusion

We have introduced a “plug-and-play” platform for fabricating fully integrated, fully printed wireless sensor nodes. The platform is implemented by placing the different components of a wireless sensing node on different faces of a cube or rectangular prism, which are joined by nonplanar RF transmission lines called radial interconnects. These components can be swapped to produce wireless sensor nodes having different functions, allowing a wide range of design goals to be met using a single platform and fabrication process. The interconnects are low-loss and broadband, offering high performance across a wide range of operating frequencies.

A demonstration of the design, testing, and fabrication of a wireless sensor node using this novel platform has been provided. First, individual components are designed and fabricated using the same additive manufacturing process as the cube platform. These components are individually characterized to ensure their performance meets design requirements. Next, components are tested together by integrating subsystems. This can be done by integrating components in fabrication, such as when the RF tag was created, or by joining individually fabricated components together with wires. After verifying the design operation, the device can be fabricated as an integrated system using the additive manufacturing process developed specifically for this platform.

Although there has been extensive development on additively manufactured components for wireless sensing nodes, research on fully integrated systems lags behind. Furthermore, a single solution that is compatible with existing developments on additively manufactured sensing components has yet to be proposed. Providing a single platform that can be used to build a wide variety of wireless sensor nodes addresses this need. It enables the rapid development of wireless sensor networks by simplifying the integration and fabrication process of wireless sensor nodes, while still offering design flexibility, so as to not limit the potential functions and features of the sensor nodes.

This platform provides a starting point for the continued production of fully integrated, fully printed wireless sensor nodes. To take full advantage of the platform, additional components must be developed using the given fabrication process. This could include sensors, antennas, power sources, and any components which add additional features to the device. Of particular interest is replacing the solar cell with an energy-harvesting system, which would allow the device to operate in environments without sufficient light to power a solar cell. Furthermore, the simple backscatter frontend could be replaced with a more complex microprocessor-based design. This would require an investigation of different methods for identifying various peripherals, as well as the development of firmware capable of implementing the “plug-and-play” approach presented here. These developments will transition the proof-of-concept approach presented here into a platform that is commercially viable and can be practically implemented.

## Methods

### Additive manufacutring fabrication process

A single layer of polypropylene (PP) is 3D printed to produce a 2D flexible substrate, and the side of the PP that was in contact with the build plate is referred to as the top. The top side of the substrate is sanded using sandpaper of grit P800, P1000, P1500, P2000, and P3000, in that order. The substrate is then subject to a UV ozone cleaning treatment for 3 min, with the top side facing up. UV ozone treatment improves the surface wetting of the substrate, which improves the smoothness of prints. All inkjet printing is done using a Dimatix 2800 printer and 2.4 pL Samba cartridges, both made by Fujifilm.

SU8, an optically transparent, photoresistive ink, is printed on the top side of the substrate. The SU8 serves as a buffer layer, further smoothing the substrate surface so that conductive SNP traces can be successfully printed. The SU8 ink is formulated with a 3:2 ratio of 2002 and 2000.5 SU8 solutions, respectively. The print head height is set to 800 um and the bed temperature is set to 39°C. After 12 layers of SU8 are printed, the SU8 is cured twice using a CL-1000 ultraviolet crosslinker. The substrate is subject to another round of UV ozone cleaning for 1 min 30 s, which improves surface wetting for the silver ink, and then is cleaned with ethanol. The total thickness of the substrate, defined as the combined thickness of the PP and cured SU8, was measured to be 0.3 mm.

Three layers of SNP ink, which in total are 1 µm thick, are deposited on top of the SU8. The print head height is raised to 1000 µm due to the additional thickness of the SU8 and the bed temperature is reduced to 35°C. After printing, the wet SNP ink is dried in an oven at 90°C for 10 min. If the ink is not visibly dry after 10 min, appearing dark gray instead of silver, the print may be left in longer. The silver should not be dried in the oven for more than 20 min to minimize the risk of substrate warping. The dry silver ink is subject to UV curing using a Xenon X-1100 and Xenon LC-912 photonic curing chamber, using two rounds of 60 pulses at 2000 V and 500 J.

### Additional fabrication steps

Many components, such as transmission lines, require a conductive ground plane for good RF transmission. This is implemented by sticking copper tape to bottom side of the substrate. Other components must be conformed to a 3D structure, which is done by securing the final 2D design to a 3D printed structure using cyanoacrylate, a type of adhesive. This support structure is also additively manufactured, as it is stereolithography (SLA) printed using FormLabs Clear resin on a Form3 SLA printer. Finally, prints requiring lumped elements have the appropriate components attached. Instead of using solder, which would melt the PP, the surface mount components are secured using silver conductive epoxy adhesive 8331D, made by M. G. Chemicals.

### Circuit design specifications

The operational amplifier used was a TSU101 and the VCO used was a LTC6907. Resistor values were as follows: R2 = 47 k$\Omega$, R3 = R4 = R5 = 7.15 k$\Omega$, R6 = R7 = 51.0 k$\Omega$, R8 = 56.2 k$\Omega$, R9 = 499.0 k$\Omega$, R10 = 5.6 k$\Omega$, and R11 = 1.91 k$\Omega$. R1 is selected according to the base resistance of the sensing element. The diode used was SMS201 Schottky diode, which has a low forward voltage drop of around 80 mV. Two 300 nH inductors were used to block DC signals from the diode.

### Measurement setup

The setup for the range measurement (Fig. 5b) uses two horn antenna to interrogate the tag. The transmitting horn antenna is connected to a signal generator, while the receiving horn antenna is connected to a spectrum analyzer. A low-noise amplifier (LNA) is placed between the receiving horn antenna and the coaxial cable connecting it to the spectrum analyzer. The tag is modulated between ON and OFF states at 10 MHz, and the power is recorded while the range is swept by physically moving the tag.

The measurement setup for the joined components test (Fig. 5c) and fully integrated device (Fig. 6b) was similar. The system is placed at a fixed distance of 1 m from a pair of horn antenna. One horn antenna is connected to a signal generator that outputs a 5.8 GHz signal, and the other is connected to a spectrum analyzer. An LNA is placed between the receiving horn antenna and spectrum analyzer to improve the sensitivity of the receiver. The modulation frequency response of the system is recorded while the ambient air temperature is increased using a heat lamp. The exact air temperature is measured using a thermocouple.

## Data Availability

The datasets used and/or analyzed during the current study available from the corresponding author on reasonable request.
